# Flow Diverting Stents for the Treatment of Complex Visceral and Renal Aneurysms—A Systematic Review

**DOI:** 10.3390/jcdd12090346

**Published:** 2025-09-11

**Authors:** Marcello Andrea Tipaldi, Elisa Zaccaria, Nicolò Ubaldi, Edoardo Massaro, Gianluigi Orgera, Tommaso Rossi, Aleksejs Zolovkins, Miltiadis Krokidis, Pasqualino Sirignano, Michele Rossi

**Affiliations:** 1 Interventional Radiology Unit, Department of Surgical and Medical Sciences and Translational Medicine, Sant’Andrea Hospital of Rome, “Sapienza” University of Rome, 00189 Rome, Italy; elisa.zaccaria@uniroma1.it (E.Z.); ubaldi.nicolo@gmail.com (N.U.); edoardomassaro72@gmail.com (E.M.); orgera.g@gmail.com (G.O.); aleksej@libero.it (A.Z.); michele.rossi@uniroma1.it (M.R.); 2 Department of Radiological Sciences, Oncology and Pathology, Policlinico Umberto I Hospital, “Sapienza” University of Rome, Viale del Policlinico 155 Sapienza, 00161 Rome, Italy; tommirossi97@hotmail.it; 3 School of Medicine, Areteion Hospital, National and Kapodistrian University of Athens, 11528 Athens, Greece; mkrokidis@hotmail.com; 4 Vascular and Endovascular Surgery Unit, Department of General and Specialistic Surgery, Sant’Andrea Hospital of Rome, “Sapienza” University of Rome, 00189 Rome, Italy; pasqualino.sirignano@uniroma1.it

**Keywords:** visceral aneurysm, visceral pseudoaneurysm, renal aneurysm, flow-diverter stent, interventional radiology

## Abstract

Flow-diverting stents (FDS) are sophisticated endovascular devices that aim to modulate blood flow and promote aneurysm thrombosis while maintaining branch vessel patency. Initially designed and developed for the treatment of intracranial aneurysms, these devices have since been applied to the peripheral circulation. However, they are still used sporadically, largely due to a lack of the scientific evidence supporting its use in visceral aneurysms. This review article aims to provide an overview of the current data on the clinical outcomes from the use of FDS in the treatment of complex visceral and renal aneurysms or pseudoaneurysms and to assess the added value of these devices.

## 1. Introduction

Visceral artery aneurysms (VAAs) and pseudoaneurysms (VAPAs) are rather uncommon however potentially life-threatening vascular pathologies, with a reported incidence rate per year ranging between 0.01% and 0.2% [1]. VAAs are generally secondary to pre-existing arteriosclerotic damage of the vessel walls and mainly due to degeneration of the tunica media, more commonly seen in older patients. On the other hand, VAPAs are typically related to local inflammation (mainly pancreatitis), dissection or vascular trauma including iatrogenic causes [2,3,4].

Splenic artery aneurysms have the highest prevalence among VAAs (60%), followed by hepatic (20–50%), superior mesenteric (6%) and celiac artery aneurysms (4%) [5]. The majority of VAAs increase in size slowly over time, but in the presence of hyper-flow conditions (as pregnancy and infections) or hypertension, the risk of growth and subsequent rupture may increase significantly [6]. The indications for treatment depend, mainly, on their location and size, with a threshold of 2 cm for most of the cases. Pseudoaneurysms, on the other hand, should be treated irrelevantly from their size, due to the higher risk of rupture [7,8,9]. Due to the lower invasiveness and morbidity in comparison to traditional open surgery, the endovascular approach is the first line treatment for VAAs and VAPAs where the endovascular expertise is available [10,11,12].

VAA can be classified into saccular, fusiform and complex. The most challenging scenario is to treat distal VAA with side-branches originating from the parent vessel and/or from the sac itself. In this case conventional treatment with coils or covered stents inevitably will cause the occlusion of one or more side-branches with consequent end-organ ischemia. This is fundamental in end-organ VAA such as kidney or mesenteric vessels where the collateral supply is insufficient or absent. Therefore, preserving both the feeding artery and the side branches is a desirable solution that was achieved with the flow-diversion techniques that were initially applied for the treatment of intracranial aneurysms and recently expanded to the peripheral circulation.

Flow-diverting stents (FDS) are structured with a greater metal surface area to redirect flow along the main vessel and minimize flow into the aneurysm, while maintaining enough flow through the porous stent to ensure branch vessels and perforators remain open [13]. They facilitate aneurysm occlusion as following: firstly, by diminishing direct jet blood flow into the aneurysm and promoting organised flow along the stent, known as central diversion [14]; secondly, by slowing the blood flow within the aneurysm, increasing stagnation, and favouring intra-aneurysmal thrombosis; and thirdly, by reducing wall stress and stagnant flow, leading to aneurysm remodeling and eventually promoting endothelialization across the aneurysm neck and arterial reconstruction [15]. Dual antiplatelet therapy adherence is required to maintain stent patency, for a duration of 1 to 6 months, followed by life-long therapy with aspirin [7]. Small differences have been observed in terms of the duration of dual antiplatelet therapy, with acetylsalicylic acid 100 mg/day and clopidogrel 75 mg/day.

Despite its appeal, the use of FDS is not widely adopted for the treatment of visceral aneurysms. One potential reason is the concern that was expressed with the first-generation devices is that the generated thrombosis may not sufficiently prevent aneurysm expansion [16]. Therefore, the existing data are limited and mainly consist of case series and short reports. A meta-analysis with data from 10 cohort studies on 220 patients was published in 2020 reporting high rates of successful side branches patency, complete aneurysms thrombosis, and high primary stent patency rate [17]. Nevertheless, the latter study includes evidence from the old generation of flow-diverting stents.

This comprehensive and systematic review aims to provide the most recent data on the efficacy and safety of FDS, and to compare the new generation of flow-diverting stents (NGFDS) with the old generation of flow-diverting stents (OGFDS) and with any other device or technique used for a flow diversion effect, i.e., the double layer nitinol stents or the Multi-Layered Bare Stent technique (MOUS) in the management of VAPAs and VAAs.

## 2. Materials and Methods

### 2.1. Search Method

This comprehensive and systematic review has been written according to the Preferred Reporting Items for Systematic Reviews and Meta-Analyses (PRISMA). Approval from the Institutional Review board was not necessary. PubMed was the database used for articles’ selection and creation of the database. The systematic review was registered on the PROSPERO database (number CRD420251121727).

### 2.2. Article Selection

The search was conducted including all articles within the years 2008 to the end of 2024 responding to the key words (Flow diverter stent OR flow diverting stent OR flow modulating stent OR neurointerventional stent OR cardiatis OR multilayer stent OR derivo OR micromesh stent OR dual-layer stent OR braided stent) AND (visceral aneurysm OR renal aneurysm). We selected those in which either a FDS was used as a therapeutic choice or any device/technique was used with a flow diversion function, exclusively for visceral arterial aneurysms and pseudoaneurysms, including renal ones with no exclusion on aneurysms’ size. Pseudoaneurysms were also included for the sake of completeness, as they have been considered in multiple studies, despite our doubts in FDS clinical appropriateness for treating these lesions at high risk of bleeding which require a prompt and definitive exclusion. All included cases present a minimum of 12 months of follow-up events as considered by the authors as a sufficient period of the time for the evaluation of the treatment. Efficacy was defined as complete exclusion of the aneurysm without reperfusion observed at the Angio-CT. Articles in which aneurysm or pseudoaneurysms were treated with classic techniques like covered stents or coils were excluded as well as those in which a combination of techniques were used. Articles with cases having <12 months follow-up were excluded, being considered a minimum time for outcome evaluation.

### 2.3. Data Collection and Organization

**Figure 1:**
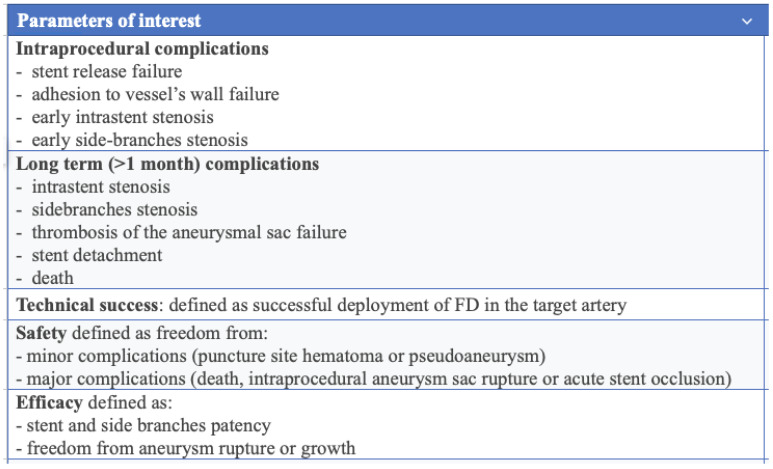
Parameters of interest.

Data from research papers were extracted as following: aneurysm location and size, type of stent used, periprocedural complications, follow-up events and therapy delivered. All data were collected with the purpose of categorizing them according to specific parameters of interest which are described in Figure 1.

Two authors independently assessed the risk of bias with the use of JBI Critical Appraisal Checklist and ROBINS1 simplified tool (Appendix A) and extracted the data. In cases of discrepancies, two senior reviewers were consulted to reach a final consensus.

## 3. Analysis and Results

**Figure 2:**
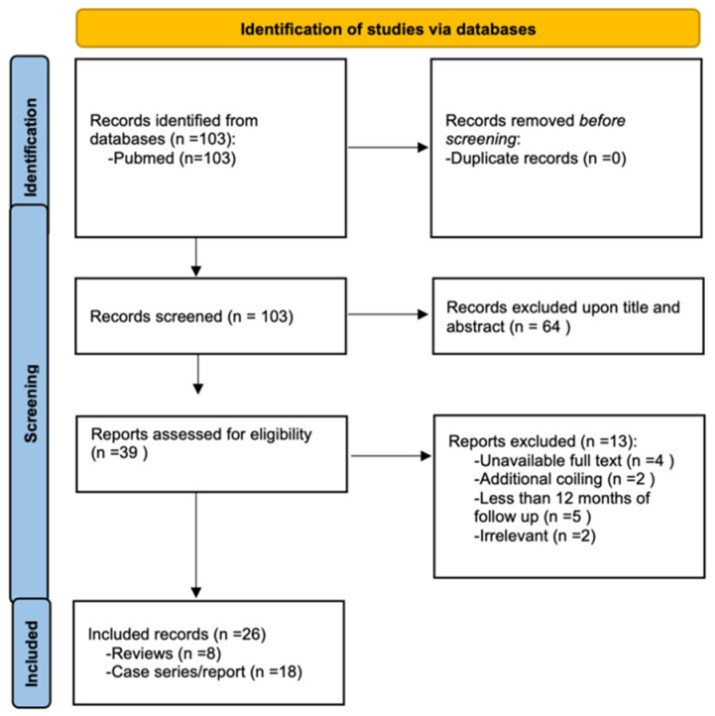
Flowchart depicting the process of study identification, screening, eligibility assessment, and inclusion in the systematic review (n = number of patients in the sample).

Out of 103 articles considered, 64 were excluded based on title and abstract not meeting the criteria. Of the remaining 39, four were excluded due to unavailable full-text, essential for scientific value. Out of the others, two were excluded as the treatment involved coil usage, and 2 did not have enough relevant data. After this selection process, 31 articles were analysed: eight reviews and 23 case reports\series; within these, five have been excluded because of less than 12 months follow up (Figure 2).

**Figure 3:**
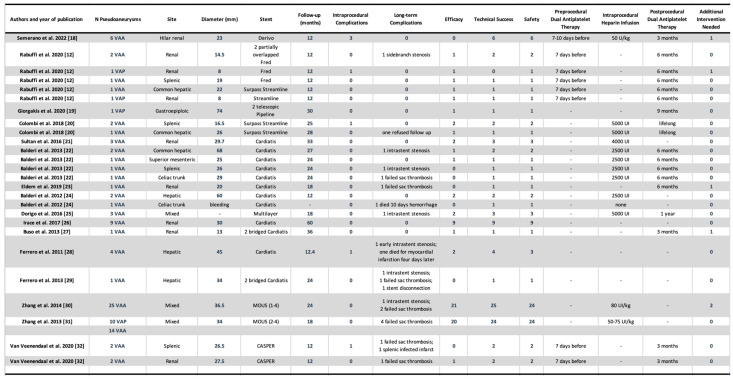
Characteristics and major outcomes of enrolled research studies [12,18,19,20,21,22,23,24,25,26,27,28,29,30,31,32].

**Figure 4:**
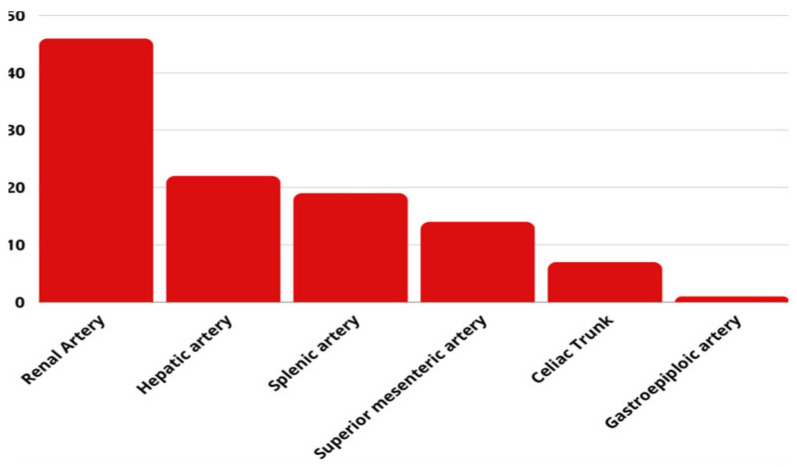
Location of aneurysms and pseudoaneurysms.

A total of 18 reports were included in this systematic review. A total of 99 patients with visceral or renal aneurysms/pseudoaneurysms were treated with a flow diverter stent or using a flow diverter effect method in this research period. Description of each enrolled study is summarized in Figure 3. A summary of the aneurysms’ location documented in the studies enrolled are shown in Figure 4.

### 3.1. Analysis on the Entire Population

In this systematic review, the major parameters analysed were: technical success, safety, and efficacy, respectively defined as shown in Figure 1.

For the use of a flow diverting option, which includes properly defined flow diverter stents and other devices or techniques to obtain a flow diverter effect, the study documented an overall efficacy rate of 78% (69.59–85.97%; CI 95%), a safety rate of 98.9% (96.99–100%; CI 95%) and a technical success of 94.9% (90.66–99.22%; CI 95%) in 99 aneurysms treated (Table 1). The mean transverse diameter of the aneurysms was 27 mm (+\− 15 mm; SD) and a mean follow-up of 21.7 months (+\− 11 mm; SD). The type (Sacciform, fusiform and complex) of aneurysms was rarely mentioned in the articles.

The population was then divided into three subgroups based on the type of flow diversion used.

### 3.2. First Group Analysis: The New Generation Flow Diverter Stent (NGFDS)

The first group includes procedures with a flow diverter stent developed in recent years: Derivo, (Derivo embolization device, Acandis, Pforzheim, Germany), Fred (MicroVention, Tustin, CA, USA), Surpass Streamline (Stryker, Tokyo, Japan), Pipeline embolization device (Medtronic, New York, NY, USA). Between 2018 and 2023 in all the reviewed articles, these flow diverters were used n 16 aneurysms out of 99 examined. The mean transverse diameter of the aneurysms treated was 16 mm (9.6–2.4; CI 95%) and a mean FU of 16 months (+\− 6.5 mm; SD). NGFDS were associated with an efficacy rate of 93.75% (82–100%; CI 95%), a safety rate of 100% (81.25–100%; CI 95%) and a technical success of 75% (54–96%; CI 95%) as shown in Table 1a.

### 3.3. Second Group Analysis: Old Generation Flow Diverter Stent (OGFDS)

The second subgroup includes cases treated with the Multilayer Flow Modulator, Cardiatis SA (Isnes, Belgium), which is a stent initially developed for aorta aneurysms then used for visceral arteries and considered as “Old Generation”. Records show that it was most used off-label in the early years for the treatment of visceral aneurysms, and in this group were selected 30 pts out of 99 from 2008 to 2019. The Cardiatis stent was associated with an efficacy rate of 66.66% (49.81–83.51%; CI 95%), a safety rate of 96.66% (90.25–100%; CI 95%) and a technical success of 100%. (90–100%; CI 95%). The mean transverse diameter of the aneurysms treated was 34 mm (28–40%; CI 95%) and a mean FU of 26 months (+\−12.9 m; SD) as shown in Table 1b.

### 3.4. Third Group Analysis: Double Layer Braided Mesh Stent and MOUS Technique (OTHERS)

The third and final group includes 53 patients in which aneurysms were treated using multilayer stents in order to achieve a flow diversion effect, such as in the case of the Casper double layer braided mesh stent or through the multiple overlapping uncovered stents (MOUS) technique.

The MOUS technique was associated with an efficacy rate of 79% (68.02–89.98%; CI 95%), a safety rate of 100% (94.34–100%; CI 95%) and a technical success of 98% (94.23–100%; CI 95%). The only case of intrastent stenosis occurred has not been considered as declared from the authors due to failed patient’s compliance to the dual antiplatelet therapy. The mean transverse diameter of the aneurysms treated was 31.12 mm (29.8–32.5; CI 95%) and a mean FU of 16 months (+\−6 months; SD), as shown in Table 1c.

### 3.5. Comparative Analysis

**Figure 5:**
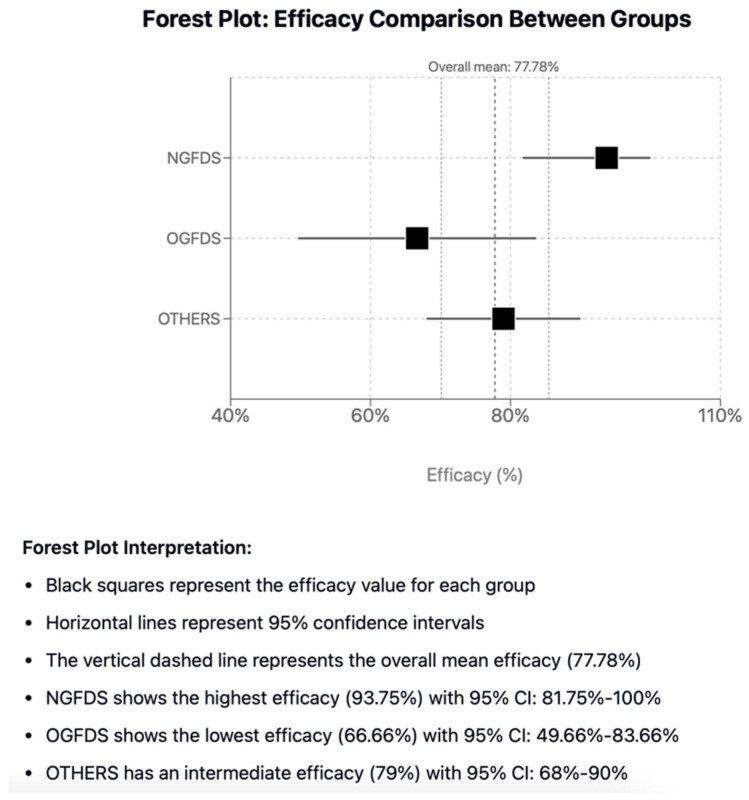
Forest Plot: efficacy comparison between groups.

The comparative analysis of efficacy between the three groups under examination (Table 2 and Figure 5, resulted in a statistically significant difference between NGFDS and OGFDS [OR = 7.5 (95% CI: 0.86–65.2); *p* = 0.048]. Meanwhile, the difference in efficacy between NGFDS and the third group was not statistically significant, nor was the difference between the latter and OGFDS [OR = 3.93 (95% CI: 0.47–32.9); *p* = 0.174 and OR= 0.52 (95% CI: 0.19–1.42); *p* = 0.207, respectively). No statistically significant differences in safety were detected between groups (Table 3); meanwhile the comparative analysis for technical success between both NGFDS and OGFDS and NGFDS and OTHERS appear statistically significant not in favor of NGFDS [OR = 0.08 (95% CI: 0.01–0.56); *p* = 0.005 and OR = 0.07 (95% CI: 0.01–0.54); *p* = 0.002, respectively) (Table 4 and Table 5).

The risk of bias assessment tools applied the comparison between NGFDS and OGFDS does not appear to be strongly influenced by differences in risk of bias between the groups, given that the weighted risk scores are similar (2.20 vs. 2.29). However, the poor management of confounding factors in all groups represents a significant limitation for conducting a meta-analysis.

## 4. Discussion

Flow-diverting stents, originally developed for treating intracranial aneurysms, have been increasingly used in managing complex visceral arterial aneurysms with side-branches [16,30] with good results [33], even though no consensus has been obtained internationally for their use, due to the lack of convincing evidence. No prospective randomized control data are available for analysis; however, a recent meta-analysis has reported high technical success rates, sac thrombosis, efficacy and safety [17]. When branching vessels originate from a visceral aneurysm, covered stents may be unsuitable implying the coiling of the branches; in such cases, FDS can help preserve distal run-off and end-organ perfusion [34]. This systematic review covers the most recent data on the use of FDS in peripheral VAA and VAPA and compares the efficacy and safety of the newest, oldest and multi-layered ones.

Flow-diverter technique arises from the device’s tight micromesh design to induce flow modulation rather than sealing it off, which depends on its porosity (the metal-free area relative to the total stent surface) and pore density (number of pores per unit area along the device-neck interface) [33]. Both porosity and pore density influence the resistance to blood flow into the aneurysmal sac, and FDS promote slow sac thrombosis whilst preserving patency of collateral branches, crucial in territories with a high risk of distal ischaemia. Moreover, NFDS are more flexible and are easier to navigate than stent-grafts and could be a favourable choice when deploying in tortuous long vessels [17,35]. The primary advantage of FDS in preserving side-branches is particularly relevant when branches originating directly from the sac supply distal vital organs, where stent-grafts or coiling would not achieve the same conservative outcomes. Preserving the flow in visceral organ may be generally advised, when possible, to avoid distal ischemic sequels. This is particularly important in the renal or mesenteric arteries where the embolization of collaterals may lead to severe clinical complications.

Hemodynamic flow studies have shown that multiple overlapping uncovered stents manage to thrombose the sac whilst keeping the branch unobstructed [36]. Zhang et al. discovered a “unique flow route” inside the thrombosed aneurysm sac that allows for branch runoff in these situations [30]. Novel thin-film nitinol flow diverters, made from patterned sheets rather than braided wires, allows for a significantly higher pore density compared to traditional braided wire flow diverters and have shown promising results in preclinical tests [37].

In this comprehensive and systematic review, the aim was to report the results of FDS in the management of VAA and VAPA and categorize enrolled studies into three groups for comparison: new generation FDS, old generation FDS, and other devices or techniques used to obtain a flow diversion effect. When considering the entire population, they were associated with significantly high, overall side-branch vessel patency (96.9%), treatment efficacy (78%) and safety (98%) and sac thrombosis (89%), which may be beneficial for sac stabilization and shrinkage [17]. Stent induced thrombosis could be related with decreasing the wall’s tensile stress and eliminating local stress concentrations [38]. Partial thrombosis has not been associated with reduced aneurysm shrinkage [30,38], although two case of partial thrombosis of the sac in the OGFDS were associated with increased size of the sac which eventually required re-intervention [23,29]. On the other hand, the 6 cases of partial thrombosis with MOUS technique did not show any increase in size nor cause of re-intervention compared to the complete thrombosis cases [30,31].

**Figure 6:**
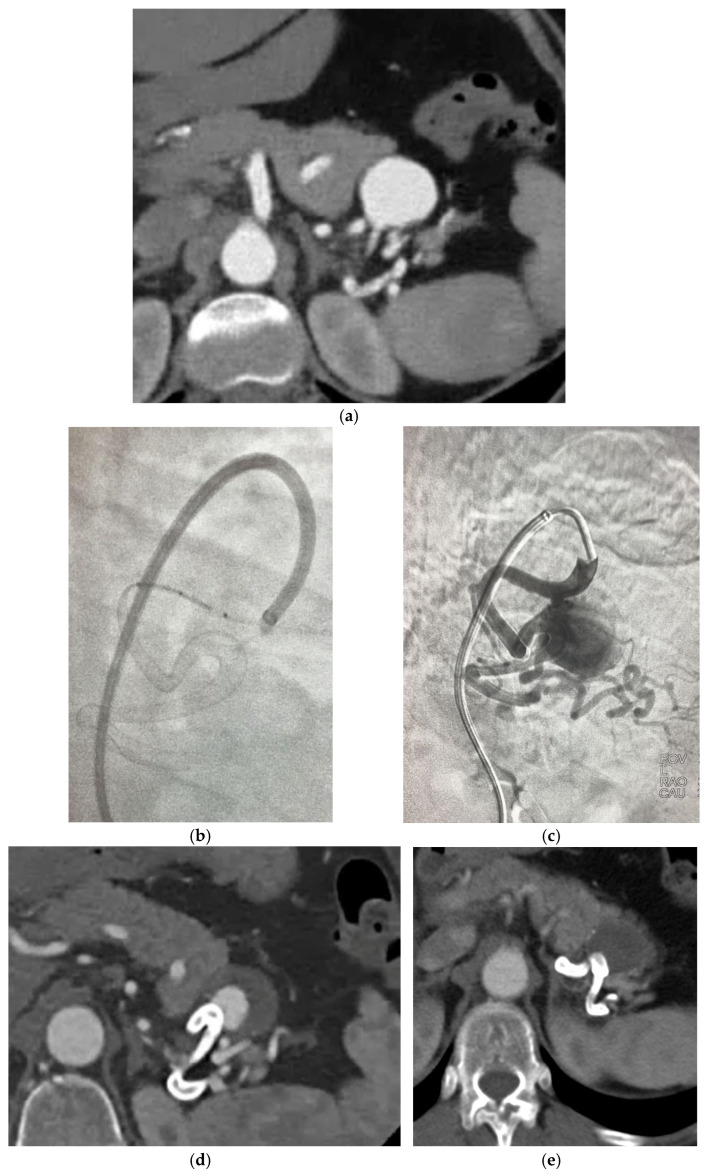
(**a**) Angio—CT shows 4-cm splenic distal artery aneurysm with three efferent vessels. (**b**,**c**) DSA showing the placement of a FD Derivo 6 × 5 cm. (**d**,**e**) Angio CT of 1 and 6 months FU with partial and total thrombosis of the sac. Patency of efferent vessels.

The new generation FDS have been associated with the highest rate of treatment efficacy (93.75%) compared to the other groups, however, despite the highest rate of intraprocedural complications (31.25%) and lowest technical success rates (75%). The cases reported having technical difficulties were due to prolapse of the stent in the aneurysm sac or to vessel dissection, 12% of which required re-intervention [18,23]. Intraprocedural issues were not seen in MOUS technique, only 1 case was observed in the use of double layer braided mesh stent which required reintervention with an additional stent positioning due to persistency of the aneurysm at 12 months CT angiogram [32] and only 1 in OGFDS Cardiatis [39], possibly due to the longer experience of doctors with these devices compared to the newer ones. The high rate of technical complications associated with the use of NGFDS can be partly explained by their predominant use in particularly complex cases, where they often represent the only available therapeutic option. This is especially true for tortuous and distal locations, where other techniques are not applicable. However, not all the articles analysed the exact location of the aneurysm, hence we could not clearly highlight this aspect, thus limiting our ability to directly correlate technical complications to the specific anatomical location of the treated aneurysms. Another issue regarding NGFDS, is the size available to date that limit their use in vessel with a dimeter > 7 mm. Figure 6a–e describe a case of NGFDS from our institution.

Stent occlusion is an issue observed in studies involving FDS [39], partly associated to failure of patient’s compliance in undergoing correct anti-platelet therapy [22,25,28,30,40]. In-stent thrombosis can occur anywhere from soon after placement to up to 24 months after the procedure. According to our data, the primary stent patency rate stands at 94.74% on average, and the only reported cases of stent thrombosis were associated with OGFDS, some of which required angioplasty [23]. The stent thrombosis was not associated with sac perfusion.

Of importance, the only case of mortality associated to the procedure is reported by Balderi et al. in a case of a bleeding VAA of the celiac trunk as the FDS was inappropriately chosen as it did not manage to limit the haemorrhage [24]. A similar case but with a successful outcome has been reported by Giorgakis et al. in the treatment of a sidewall bleeding VAPA of right Gastroepiploic artery with the use of two Pipeline stents embolization devices with a telescoping method in a patient after pancreatectomy, permitting to preserve the main arterial supply to the gastric conduit [19].

The analysis of this systemic review data has limitations that must be accounted for. Firstly, the data retrieved were from small cohort, non-comparative, retrospective studies which lacked randomization, mainly due to the low prevalence of VAA\VAPAs. Furthermore, inconsistencies in the reporting standards of these studies made data retrieval challenging and imprecise for several parameters. Secondly, it was not possible to directly compare the different groups of stents based on the location of artery deployment, calibre, or underlying atheromatous disease. Moreover, many cases reported have not specified the patient’s adherence to anti-platelet therapy, which is very important in the management of these pathologies. For completeness of this systematic review we have included the analysis of FDS in VAPA’s, although we believe that these lesions have a high risk of bleeding and should therefore be treated with a fast and complete exclusion with front-and-backdoor coiling or with covered stenting. Lastly, older generation stents have been used for visceral aneurysms for over 10 years, whereas new generation stents have only been reported in cases from the past five years, creating a bias in comparing intraprocedural success and complications as doctors deploying older stents might have had more experience.

## 5. Conclusions

FDS represent a valid alternative for the management of complex peripheral VAAs and VAPAs, where other endovascular and surgical techniques are not feasible. Regarding the comparison between the flow diversion stent techniques described in the literature, it seems that the NGFDS are superior in terms of efficacy, despite less favourable data on their technical success and intra-procedural complications. Long-term complications were not present in new generation devices, promoting their role in avoiding intrastent thrombosis or incomplete sac exclusion, whilst preserving side-branches vessels.

To date, a major limitation in the use of flow diverters is their high cost. However, given the excellent results demonstrated by their application, increased use in the future may lead to cost reduction as happened with several devices that were transferred from neuro to peripheral intervention. Furthermore, dedicated large size devices are also now available overcoming a limitation of their past use. Further studies in this area may be required to shed light is this continuously developing field.

## Figures and Tables

**Table 1:** Details of the procedural complications and success parameters in the entire population. (**a**) Details of the procedural complications and success parameters in the first group NGFDS. (**b**) Details of the procedural complications and success parameters in the second group OGFDS. (**c**) Details of the procedural complications and success parameters in the third group OTHERS.

SUCCESS PARAMETERS	N° Procedures	Percentage on Total	95% Confidence Interval
Technical success	94	94.94%	90.66–99.22%
Safety	98	98.98%	96.99–100%
Efficacy	77	78%	69.59–85.97%
**COMPLICATIONS**	**N° Procedures**	**Percentage on Total**	**95% CI**
Intraprocedural	7	7.00%	2.01–12.13%
Long term (>1 month)	21	21%	13.17–29.25%
- Intrastent stenosis	5	5.05%	0.73–9.37%
- Side branches stenosis	3	3.03%	0–6.42%
- Failed sac thrombosis	11	11.00%	4.93–17.29%
- Splenic septic infarct	1	1.01%	0–2.99%
- Stent disconnection	1	1.01%	0–2.99%
- Mortality	1	1.01%	0–2.99%
Additional interventions needed	6	6.06%	1.35–10.77%
(**a**)			
**SUCCESS PARAMETERS**	**N° procedures**	**Percentage on total**	**95% Confidence Interval**
Technical success	12	75%	54–96%
Safety	16	100%	81.25–100%
Efficacy	15	93.75%	82–100%
	**N° Procedures**	**Percentage on Total**	**95**
Intraprocedural	5	31.25%	9–54%
Long term (>1 month)	1	6.25%	0–18%
- Intrastent stenosis	0	0%	0–18.75%
- Side branches stenosis	1	6.25%	0–18%
- Failed sac thrombosis	0	0%	0–18.75%
- Mortality	0	0%	0–18.75%
Additional interventions needed	2	12.50%	0–29%
(**b**)			
**SUCCESS PARAMETERS**	**N° Procedures**	**Percentage on Total**	**95% CI**
Technical success	30	100%	90–100%
Safety	29	96.66%	90.25–100%
Efficacy	20	66.66%	49.81–83.51%
**COMPLICATIONS**	**N° procedures**	**Percentage on total**	**95% CI**
Intraprocedural	1	3.33%	0–9.74%
Long term (>1 month)	10	33.33%	16.48–50.18%
- Intrastent stenosis	5	16.66%	3.33–29.99%
- Side branches stenosis	0	0.00%	0–10%
- Failed sac thrombosis	3	10%	0–20.74%
- Stent disconnection	1	3.33%	0–9.74%
- Mortality	1	3.33%	0–9.74%
Additional interventions needed	2	6.66%	0–15.57%
(**c**)			
**SUCCESS PARAMETERS**	**N° Procedures**	**Percentage on Total**	**95% CI**
Technical success	52	98%	94.23–100%
Safety	53	100.00%	94.34–100%
Efficacy	42	79.00%	68.02–89.98%
**COMPLICATIONS**	**N° Procedures**	**Percentage on Total**	**95% CI**
Intraprocedural	1	2.00%	0–5.77%
Long term (>1 month)	11	20.00%	9.83–31.67%
- Intrastent stenosis	0	0.00%	0–5.66%
- Side branches stenosis	2	4.08%	0–8.89%
- Failed sac thrombosis	8	15%	5.45–24.73%
- Septic infarct	1	2%	0–5.56%
- Mortality	0	0.00%	0–5.66%
Additional interventions needed	2	4.00%	0–8.89%

**Table 2:** Overall efficacy rates in the three groups.

GROUP	Efficacy (%)	CI 95%	n	Total
NGFDS	93.75	81.75–100%	15	16
OGFDS	66.66	49.66–83.66%	20	30
OTHERS	79	68–90%	42	53
TOTAL	77.78	70.1–85.46%	77	99

**Table 3:** Overall safety rates in the three groups.

GROUP	Safety (%)	CI 95%	n	Total
NGFDS	100	81.25–100%	16	16
OGFDS	96.66	89.97–100%	29	30
OTHERS	100	94.34–100%	53	53
TOTALE	98.99	97.01–100%	98	99

**Table 4:** Overall technical success rates in the three groups.

GROUP	Technical Success	CI 95%	n	Total
NGFDS	75	54–96%	12	16
OGFDS	100	90–100%	30	30
OTHERS	98	94–100%	52	53
TOTAL	94.95	91–99%	94	99

**Table 5:** Comparative analysis for technical success.

COMPARISON	OR	CI 95%	*p*-Value
NGFDS vs. OGFDS	0.08	0.01–0.56	0.005
NGFDS vs. OTHERS	0.07	0.01–0.54	0.002

## Data Availability

PubMed, research (Flow diverter stent OR flow diverting stent OR flow modulating stent OR neurointerventional stent OR cardiatis OR multilayer stent OR derivo OR micromesh stent OR dual-layer stent OR braided stent) AND (visceral aneurysm OR renal aneurysm).

## Abbreviations

The following abbreviations are used in this manuscript:

FDS	Flow-diverting stent
VAAs	Visceral artery aneurysms
VAPAs	Visceral artery pseudoaneurysms
NGFDS	New generation of flow-diverting stents
OGFDS	Old generation of flow-diverting stents
MOUS	Multi-Layered Bare Stent technique

## Appendix A. Risk of Bias Assessment

The risk of bias analysis was applied to 10 of the 18 studies as they were the most representative for each of the three intervention groups (NGFDS, OGFDS and OTHERS), with the largest number of patients and influence on the results. Furthermore, only these 10 studies had sufficient methodological details available for a complete risk of bias assessment according to the JBI and ROBINS-I criteria.

JBI Critical Appraisal

The assessment was conducted using the following criteria based on the JBI Critical Appraisal Checklist:

1. Clearly defined inclusion criteria
2. Condition measured in a standardized and valid manner
3. Identification of confounding factors
4. Strategies to manage confounding factors
5. Outcomes measured in a valid and reliable manner
6. Appropriate follow-up
7. Loss to follow-up < 20%
8. Appropriate statistical analysis
9. Clearly defined study methodology

**Table A1:** Results of the assessment for individual studies.

Group	Study	Author	n	q1	q2	q3	q4	q5	q6	q7	q8	q9	Overall Risk
NGFDS	1	Rabuffi et al., 2020 [12]	6	Y	Y	U	U	Y	Y	Y	U	Y	Unclear
NGFDS	2	Semeraro et al., 2022 [18]	8	Y	Y	N	U	Y	Y	Y	Y	Y	Moderate
NGFDS	3	Giorgakis et al., 2020 [19]	1	Y	Y	N	N	Y	U	Y	NA	Y	Moderate
OGFDS	4	Ferrero et al., 2011 [28]	2	Y	Y	N	N	Y	Y	Y	U	Y	Moderate
OGFDS	5	Ferrero et al., 2013 [29]	1	Y	Y	N	N	Y	Y	Y	NA	Y	Moderate
OGFDS	6	Balderi et al., 2013 [22]	5	Y	Y	N	U	Y	Y	Y	U	Y	Moderate
OGFDS	7	Ferrero et al., 2011 (other) [39]	11	Y	Y	U	N	Y	Y	U	U	Y	Unclear
OTHERS	8	Zhang et al., 2014 [30]	38	Y	Y	U	U	Y	Y	Y	Y	Y	Moderate
OTHERS	9	Zhang et al., 2013 [31]	10	Y	Y	U	N	Y	Y	Y	Y	Y	Moderate
OTHERS	10	Van Veenendaal et al., 2020 [32]	2	Y	Y	N	U	Y	Y	Y	NA	Y	Moderate

Legend: Y = Yes (low risk), N = No (high risk), U = Unclear (unclear risk), NA = Not applicable.

**Table A2:** Summary by study group.

Group	Total Studies	Low Risk (%)	Moderate Risk (%)	High Risk (%)	Unclear Risk (%)
NGFDS	3	0 (0.0%)	2 (66.7%)	0 (0.0%)	1 (33.3%)
OGFDS	4	0 (0.0%)	3 (75.0%)	0 (0.0%)	1 (25.0%)
OTHERS	3	0 (0.0%)	3 (100.0%)	0 (0.0%)	0 (0.0%)

**Table A3:** Analysis of critical risk domains.

Domain	Criterion	Low Risk (%)	High Risk (%)	Unclear Risk (%)
q1	Clearly defined inclusion criteria	10 (100.0%)	0 (0.0%)	0 (0.0%)
q2	Condition measured in a standardized and valid manner	10 (100.0%)	0 (0.0%)	0 (0.0%)
q3	Identification of confounding factors	0 (0.0%)	6 (60.0%)	4 (40.0%)
q4	Strategies to manage confounding factors	0 (0.0%)	5 (50.0%)	5 (50.0%)
q5	Outcomes measured in a valid and reliable manner	10 (100.0%)	0 (0.0%)	0 (0.0%)
q6	Appropriate follow-up	9 (90.0%)	0 (0.0%)	1 (10.0%)
q7	Loss to follow-up < 20%	9 (90.0%)	0 (0.0%)	1 (10.0%)
q8	Appropriate statistical analysis	3 (42.9%)	0 (0.0%)	4 (57.1%)
q9	Clearly defined study methodology	10 (100.0%)	0 (0.0%)	0 (0.0%)

**Simplified ROBINS-I Assessment**

ROBINS-I (Risk Of Bias In Non-randomized Studies—of Interventions) is a tool specifically designed to assess the risk of bias in non-randomized studies that compare the effects of two or more interventions. A simplified version was applied to the studies included in this review.

ROBINS-I Domains Assessed

1. Confounding
2. Selection of participants
3. Classification of interventions
4. Deviations from intended interventions
5. Missing data
6. Measurement of outcomes
7. Selection of reported results

**Table A4:** Results of the ROBINS-I assessment.

Study	Author	Confounding	Selection	Classification	Deviations	Missing Data	Outcome Measurement	Selection of Results	Overall Risk
1	Rabuffi et al., 2020 [12]	Moderate	Low	Low	Low	Low	Low	Moderate	Moderate
2	Semeraro et al., 2022 [18]	Serious	Low	Low	Low	Low	Low	Low	Serious
3	Giorgakis et al., 2020 [19]	Serious	Low	Low	Low	Moderate	Low	Low	Serious
4	Ferrero et al., 2011 [28]	Serious	Low	Low	Low	Low	Low	Moderate	Serious
5	Ferrero et al., 2013 [29]	Serious	Low	Low	Low	Low	Low	Low	Serious
6	Balderi et al., 2013 [22]	Serious	Low	Low	Low	Low	Low	Moderate	Serious
7	Ferrero et al., 2011 (other) [39]	Serious	Low	Low	Low	Moderate	Low	Moderate	Serious
8	Zhang et al., 2014 [30]	Moderate	Low	Low	Low	Low	Low	Low	Moderate
9	Zhang et al., 2013 [31]	Serious	Low	Low	Low	Low	Low	Low	Serious
10	Van Veenendaal et al., 2020 [32]	Serious	Low	Low	Low	Low	Low	Low	Serious

**Table A5:** Weighted risk of bias by group.

Comparison of JBI and ROBINS-I Assessments				
Assessment	Low Risk	Moderate/Unclear	High/Serious	Total
JBI	0 (0.0%)	10 (100.0%)	0 (0.0%)	10 (100%)
ROBINS-I	0 (0.0%)	2 (20.0%)	8 (80.0%)	10 (100%)
**Impact of Risk of Bias on Study Results**				
Group	Efficacy (%)	Safety (%)	Technical Success (%)	Weighted Risk of Bias
NGFDS	93.75	100.00	75.00	2.20
OGFDS	66.66	96.66	100.00	2.29
OTHERS	83.67	100.00	98.00	2.00

**Interpretation of the Impact on Results**

The comparison between NGFDS and OGFDS does not appear to be strongly influenced by differences in risk of bias between the groups, given that the weighted risk scores are similar (2.20 vs. 2.29). However, the poor management of confounding factors in all groups represents a significant limitation for conducting a meta-analysis.
